# Liver Tumor Segmentation in CT Scans Using Modified SegNet

**DOI:** 10.3390/s20051516

**Published:** 2020-03-10

**Authors:** Sultan Almotairi, Ghada Kareem, Mohamed Aouf, Badr Almutairi, Mohammed A.-M. Salem

**Affiliations:** 1 Department of Natural and Applied Sciences, Faculty of Community College, Majmaah University, Majmaah 11952, Saudi Arabia; almotairi@mu.edu.sa; 2 Department of Biomedical Engineering, Higher Technological Institute, 10th Ramadan City 44629, Egypt; ghadakareem5@gmail.com (G.K.); maoufmedical@yahoo.com (M.A.); 3 Department of Information Technology, College of Computer Sciences and Information Technology College, Majmaah University, Al-Majmaah 11952, Saudi Arabia; b.algoian@mu.edu.sa; 4 Faculty of Computer and Information Sciences, Ain Shams University, Cairo 11566, Egypt; 5 Faculty of Media Engineering & amp; Technology, German University in Cairo, Cairo 11835, Egypt

**Keywords:** deep learning, CT images, convolutional neural networks, hepatic cancer

## Abstract

The main cause of death related to cancer worldwide is from hepatic cancer. Detection of hepatic cancer early using computed tomography (CT) could prevent millions of patients’ death every year. However, reading hundreds or even tens of those CT scans is an enormous burden for radiologists. Therefore, there is an immediate need is to read, detect, and evaluate CT scans automatically, quickly, and accurately. However, liver segmentation and extraction from the CT scans is a bottleneck for any system, and is still a challenging problem. In this work, a deep learning-based technique that was proposed for semantic pixel-wise classification of road scenes is adopted and modified to fit liver CT segmentation and classification. The architecture of the deep convolutional encoder–decoder is named SegNet, and consists of a hierarchical correspondence of encode–decoder layers. The proposed architecture was tested on a standard dataset for liver CT scans and achieved tumor accuracy of up to 99.9% in the training phase.

## 1. Introduction

The liver is the largest organ, located underneath the right ribs and below the lung base. It has a role in digesting food [1,2]. It is responsible for filtering blood cells, processing and storing nutrients, and converting some these nutrients into energy; it also breaks down toxic agents [3,4]. There are two main hepatic lobes, the left and right lobes. When the liver is viewed from the undersurface, there are two more lobes, the quadrate and caudate lobes [5].

Hepatocellular carcinoma (HCC) [6] may occur when the liver cells begin to grow out of control and can spread to other areas in the body. Primary hepatic malignancies develop when there is abnormal behavior of the cells [7]. 

Liver cancer has been reported to be the second most frequent cancer to cause death in men, and sixth for women. About 750,000 people got diagnosed with liver cancer in 2008, 696,000 of which died from it. Globally, the rate of infection of males is twice than that of females [8]. Liver cancer can be developed from viral hepatitis, which is much more problematic. According to the World Health Organization, WHO, about 1.45 million deaths a year occur because of this infection [9]. In 2015, Egypt was named as the country with the highest rate of adults infected by viral hepatitis C (HCV), at 7% [9]. Because the treatment could not be reached by all infected people, the Egypt’s government launched a “100 Million *Seha*” (*seha* is an Arabic word meaning “health”) national campaign between October 2018 and April 2019. At the end of March 2019, around 35 million people had been examined for HCV [10]. 

Primary hepatic malignancy is more prevalent in Southeast Asia and Africa than in the United States [11,12]. The reported survival rate is generally 18%. However, survival rates rely on the stage of disease at the time of diagnosis [13].

Primary hepatic malignancy is diagnosed by clinical, laboratory, and imaging tests, including ultrasound scans, magnetic resonance imaging (MRI) scans, and computed tomography (CT) scans [14]. A CT scan utilizes radiation to capture detailed images around the body from different angles, including sagittal, coronal, and axial images. It shows organs, bones, and soft tissues; the information is then processed by the computer to create images, usually in DICOM format [15]. Quite often, the examination requires intravenous injection of contrast material. The scans can help to differentiate malignant lesions from acute infection, chronic inflammation, fibrosis, and cirrhosis [16]. 

**Figure 1:**
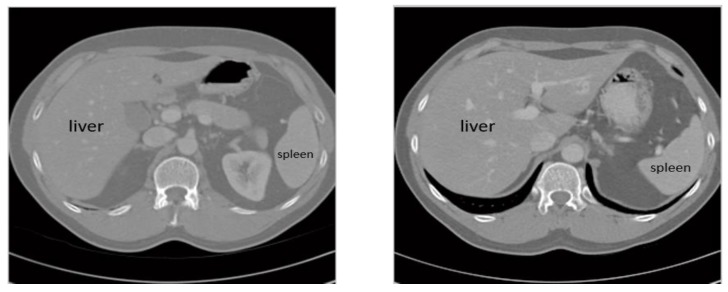
Example of the similarity in gray levels between the liver and the spleen in computed tomography (CT) images. Imported from the Medical Image Computing and Computer Assisted Intervention (MICCAI) SLIVER07 workshop datasets [24,25].

Staging of hepatic malignancies depends on the size and location of the malignancy [16,17]. Hence, it is important to develop an automatic procedure to detect and extract the cancer region from the CT scan accurately. Image segmentation is the process of partitioning the liver region in the CT scan into regions, where each region represents a semantic part of the liver [18,19]. This is a fundamental step to support the diagnosis by radiologists, and a fundamental step to create automatic computer-aided diagnosis (CAD) systems [20,21,22]. CT scans of the liver are usually interpreted by manual or semi-manual techniques, but these techniques are subjective, expensive, time-consuming, and highly error prone. Figure 1 shows an example where the gray level intensities of the liver and the spleen are too similar to be differentiated by the naked eye. To overcome these obstacles and improve the quality of liver tumors’ diagnosis, multiple computer-aided methods have been developed. However, these systems have not been that great at the segmentation of the liver and lesions due to multiple challenges, such as the low contrast between the liver and neighboring organs and between the liver and tumors, different contrast levels in tumors, variation in the numbers and sizes of tumors, tissues’ abnormalities, and irregular tumor growth in response to medical treatment. Therefore, a new approach must be used to overcome these obstacles [23].

In this work, a review of wide variety of recent publications of image analysis for liver malignancy segmentation is introduced. In recent years, extensive research has depended on supervised learning methods. The supervised method use inputs labeled to train a model for a specific task—liver or tumor segmentation, in this case. On top of these learning methods are the deep learning methods [26,27]. There are many different models of deep learning that have been introduced, such as stacked auto-encoder (SAE), deep belief nets (DBN), convolutional neural networks (CNNs), and Deep Boltzmann Machines (DBM) [28,29,30,31]. The superiority of the deep learning models in terms of accuracy has been established. However, it is still a challenge to find proper training dataset, which should be huge in size and prepared by experts.

CNNs are considered the best of deep learning methods used. Elshaer et al. [13] reduced the computation time of a large number of slices by using two trained deep CNN models. The first model was used to get the liver region, and the second model was used for avoiding fogginess from image re-sampling and for avoiding missed small lesions. 

Wen Li et al. [28] utilized a convolutional neural network (CNN) that uses image patches. It considers an image patch for each pixel, such that the pixel of interest is in the center of that patch. The patches are divided into normal or tumor liver tissue. If the patch contains at least 50 percent or more of tumor tissue, the patch is labeled as a positive sample. The reported accuracy reached about 80.6%. The work presented in [12,13] reported s more than 94% accuracy rate for classifying the images either as normal or abnormal if the image showed a liver with tumor regions. The CNN model has different architectures—i.e., Alex Net, VGG-Net, ResNet, etc. [32,33,34]. The work presented by Bellver et al. [5] used VGG-16 architecture as the base network in their work. Other work [11,16,29,32,35,36] has used two-dimensional (2D) U-Net, which is designed mainly for medical image segmentation.

The main objective of this work is to present a novel segmentation technique for liver cross-sectional CT scans based on a deep learning model that has proven successful in image segmentation for scene understanding, namely SegNet [37]. Memory and performance efficiency are the main advantages of this architecture over the other models. The model has been modified to fit two-class classification tasks.

The paper is organized as follows. In the next section, a review is presented on recent segmentation approaches for the liver and lesions in CT images, as well as a short introduction to the basic concepts addressed in this work. Section 3 presents the proposed method and the experimental dataset. Experimental results are presented in Section 4. Finally, conclusions are presented and discussed in Section 5.

## 2. Basic Concepts

Convolutional neural networks are similar to traditional neural networks [20,38,39]. A convolutional neural network (CNN) includes one or more layers of convolutional, fully connected, pooling, or fully connected and rectified linear unit (ReLU) layers. Generally, as the network becomes deeper with many more parameters, the accuracy of the results increases, but it also becomes more computationally complex. 

**Figure 2:**
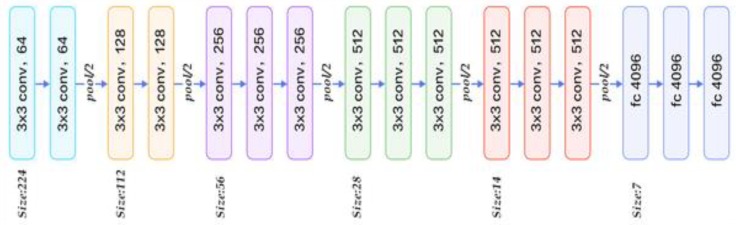
VGG-16 network architecture [44].

Recently, CNN models have been used widely in image classification for different applications [20,34,40,41,42] or to extract features from the convolutional layers before or after the down sampling layers [41,43]. However, the architectures discussed above are not suitable for image segmentation or pixel-wise classifications. VGG-16 network architecture [44] is a type of CNN model. The network includes 41 layers. There are 16 layers with learnable weights: there are 13 convolutional layers and three fully connected layers. Figure 2 shows the architecture of VGG-16 as introduced by Simonyan and Zisserman [44].

Most pixel-wise classification network architectures are of encoder–decoder architecture, where the encoder part is the VGG-16 model. The encoder gradually decreases the spatial dimension of the images with pooling layers; however, the decoder retrieves the details of the object and spatial dimensions for fast and precise segmentation of images. U-Net [45,46] is a convolutional encoder-decoder network used widely for semantic image segmentation. It is interesting because it applies a fully convolutional network architecture for medical images. However, it is very time- and memory-consuming.

**Figure 3:**
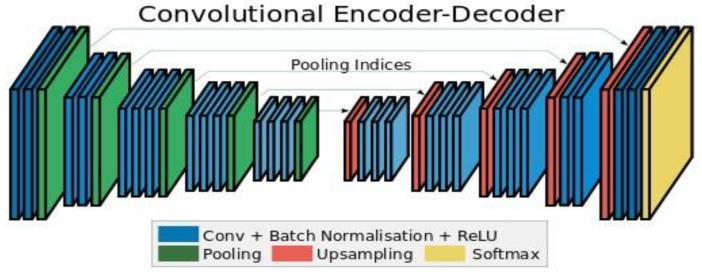
SegNet network architecture [37].

The semantic image segmentation approach uses the predetermined weights of the pertained VGG-16 network [45]. Badrinarayanan et al. [37], have proposed an encoder–decoder deep network, named SegNet, for scene understanding applications tested on road and indoor scenes. The main parts of the core trainable segmentation engine are an encoder network, a decoder network, and a pixel-wise classification layer. The architecture of the encoder network is similar to the 13 convolutional layers in the VGG-16 network. The function of the decoder network is mapping the features of encoder with low to full-input resolution feature maps for pixel-wise classification. Figure 3 shows a simple illustration of the SegNet model during the down sampling (max-pooling or subsampling layers) of the encoder part. Instead of transferring the pixel values to the decoder, the indices of the chosen pixel are saved and synchronized with the decoder for the up-sampling process. In SegNet, more shortcut connections are presented. The indices are copied from max pooling instead of copying the features of encoder, such as in FCN [47], so the memory and performance of SegNet is much more efficient than FCN and U-Net. 

## 3. Materials and Method

This section discusses the steps and the implementation of the proposed method for segmentation of a liver tumor. The proposed method follows the conventional pattern recognition scheme: preprocessing, feature extraction and classification, and post-processing.

### 3.1. Dataset

The 3D-IRCADb-01 database is composed of three-dimensional (3D) CT-scans of 20 different patients (10 females and 10 males), with hepatic tumors in 15 of those cases. Each image has a resolution of 512 × 512 width and height. The depth or the number of slices per patient ranges between 74 and 260. Along with patient images in DICOM format, labeled images and mask images are given that could be used as ground truth for the segmentation process. The place of tumors is exposed by Couinaud segmentation [48]. This shows the main difficulties in segmentation of the liver via software [49].

### 3.2. Image Preprocessing

In the preprocessing steps, the DICOM CT images were subject to file format conversion to portable network graphics (PNG). The PNG file format was chosen to preserve the image quality, as it is a lossless format. In DICOM format, the pixel values are in Hounsfield, in the range [−1000, 4000]. In this format, the images cannot be displayed, and many image processing operations will fail. Therefore, the color depth conversion, and hence the range of the pixel’s values mapping to the positive 1 byte integer, is necessary. The mapping is done according to the following formula:

(1) $$g=\frac{h-m_{1}}{m_{2}-m_{1}}*255$$

where h is the pixel value in Hounsfield, g is the corresponding predicted gray level value, and $m_{1}$ and $m_{2}$ are the minimum and maximum of the Hounsfield range, respectively.

**Figure 4:**
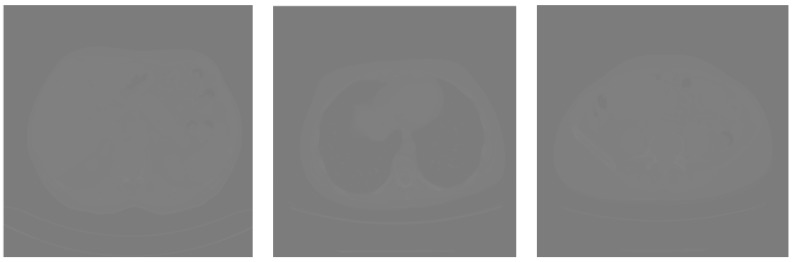
Raw CT slices in DICOM format for three different patients imported from the IRCAD dataset [50].

The second step is to put the images in an acceptable format for the SegNet model [37]. The images have been converted to three channels, similar to the RGB color space, by simply duplicating the slice in each channel and resizing each to be the dimension 360 × 480 × 3. Figure 4 shows three samples of the input images before color depth correction. The images in this format have too low contrast and are not suitable for use by the deep learning model.

In order to increase the performance of the system, the training images were subject to data augmentation, where the images are transformed by a set of affine transformations, such as flipping, rotation, and mirroring, as well as augmenting the color values [38,51,52]. Perez et al. [53] discuss the effectiveness of data augmentation on the classification results when deep learning is used, and showed that the traditional augmentation techniques can improve the results by about 7%.

### 3.3. Training and Classification 

The goodness of CNN features was compared to other traditional feature extraction methods, such as LBP, GLCM, Wavelet and Spectral. The feature extractors, which give good performance in comparison with the other texture extractor features, are a CNN. CNN training consumes some time; however, features can be extracted from the trained convolutional network, compared to other complex textural methods. CNNs have proven to be effective in classification tasks [26]. The training data and data augmentation are combined by reading batches of training data, applying data augmentation, and sending the augmented data to the training algorithm. The training is started by taking the data source, which contains the training images, pixel labels, and their augmentation forms.

## 4. Experimental Results

### 4.1. Evaluation Metrics

The output results of classification were compared against the ground truth given by the dataset. The comparison was done on a pixel-to-pixel basis. To evaluate the results, we applied the evaluation metrics given below. Table 1 represent the confusion matrix for binary class classification.

Out of the confusion matrix, some important metrics were computed, such as

1. Overall Accuracy: this represents the percentage of correctly classified pixels to the whole number of pixels. This could be formulated as in Equation (2):

(2) $$accuracy=\frac{TN+TP}{TN+TP+FN+FP}$$

while the mean accuracy is the mean of accuracies reported across the different testing folds. 

2. Recall (Re) or true positive rate (TPR): this represents the capability of the system to correctly detect tumor pixels relative to the total number of true tumor pixels, as formulated in Equation (3):

(3) $$Re=\frac{TP}{TP+FN}$$

3. Specificity of the true negative rate (TNR): this represents the rate of the correctly detected background or normal tissue, as formulated in Equation (4):

(4) $$Specificity=\;\frac{TN}{TN+FP\;}$$

Since most of image is normal or background, the percentage of global accuracy is significantly influenced by the TNR. Therefore, some other measures for the tumor class are computed. 

4. Intersection over union (IoU): this is the ratio of correctly classified pixels relative to the union of predicted and actual number of pixels for the same class. Equation (5) shows the formulation of the IoU:

(5) $$IoU=\frac{TP}{TP+FP+FN}$$

5. Precision (Pr): this measures the trust in the predicted positive class, i.e., prediction of a tumor. It is formulated as in Equation (6):

(6) $$Pr=\frac{TP}{TP+FP}$$

6. F1 score (F1): this is a harmonic mean of recall (true positive rate) and precision, as formulated in Equation (7). It measures whether a point on the predicted boundary has a match on the ground truth boundary or not:

(7) $$F1=\frac{2\left(Pr*Re\right)}{\left(Pr+Re\right)}$$

### 4.2. Data Set and Preprocessing

As mentioned before, the dataset used to test the proposed algorithm is 3D-IRCADb. The 3D-IRACDb dataset is offered by the French Research Institute against Digestive Tract, or IRCAD [50]. It has two subsets: the first one, 3DIRACDb-01, is the one appropriate for liver tumor segmentation. This subset consists of publicly available 3D CT scans of 20 patients, half of them for women patients and half for men, with hepatic tumors in 75% of the cases. All the scans are available in DICOM format with axial dimensions of 512 × 512. For each case, tens of 2D images are available, together with labeled images and masked images prepared by radiologists. In this work, we have considered all 15 cases with a total of 2063 images for training and testing. The dataset is used widely and recently, as in [54,55,56,57].

**Figure 5:**
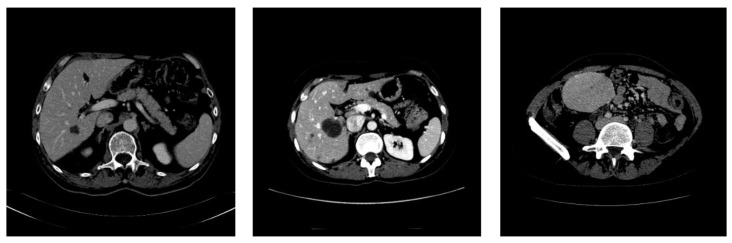
Samples of the slices after color range mapping to [0, 255]. The three images correspond respectively to the images in Figure 4.

**Figure 6:**
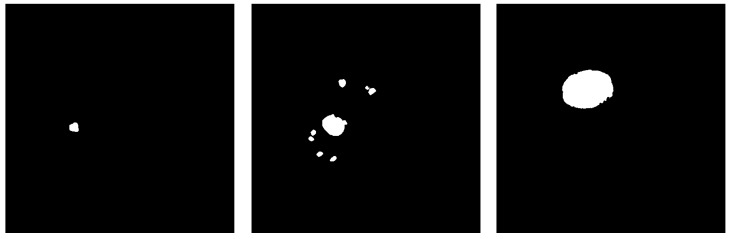
Liver tumor labeled images from the ground truth given by the dataset. The three images correspond respectively to the images in Figure 4 and Figure 5.

All image slices were subject to preprocessing, as discussed above. The labeled images provided by the dataset are preprocessed by the same procedure, except the step of range mapping, since they are given as binary images in the range [0,255]. Figure 5 and Figure 6 show the examples of the preprocessing steps on input images. Associated with the input (patient) images are the labeled images, which are labeled by experts and are fed to the system as ground truth for the segmentation process. 

### 4.3. Training and Classification

Three of the 15 cases of the dataset were used for testing and evaluation, with a total of 475 images. Among these, 454 images were used for training and validation, and 45 images were used for testing.

The first training and testing experiments were carried out using the U-Net model in [45]. The U-Net model is trained to perform semantic segmentation on medical images. It is based on VGG-16, as discussed before. The results were near perfect to extract the liver region. However, it failed completely when tested to extract the tumor regions from the image. In this case, the tumor region was almost missed or predicted as others. 

**Figure 7:**
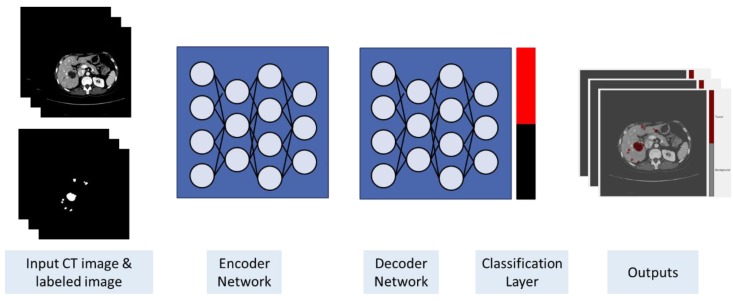
The proposed architecture.

The proposed architecture is based on the SegNet model [37], which is an encoder network, and a corresponding decoder network connected to a 2D multi-classification layer for pixel-based semantic segmentation. However, the final classification layer was replaced by 2D binary classification. The VGG-16 trained model was imported for the encoder part. Figure 7 shows an illustration of the proposed network architecture. To improve the training, class weighting was used to balance the classes and calculate the median frequency class weights. 

For testing, a semantic segmentation was returned from the input image with the classification scores for each categorical label, in order to run the network for one image from test set. 

### 4.4. Testing and Evaluation 

The proposed method was trained on a machine with NVIDIA GTX 1050 4GB RAM GPU on an Intel Core i7-7700HQ 2.20 GHz 16 GB RAM, and developed with MATLAB 2018b software, which offers a Neural Network Toolbox and an Image Processing Toolbox.

**Figure 8:**
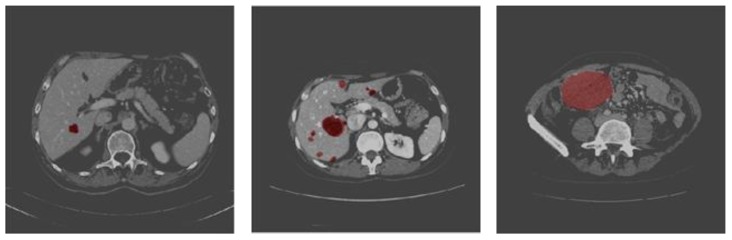
Samples of the results of testing. The predicted tumor position is highlighted with the red color.

The images of the tested cases were divided randomly into two groups for training and testing by the ratio 9:1. The results of the training are normally higher than that achieved by testing. Figure 8 shows three samples of testing output, where the resulted binary segmentation is augmented on the input gray-level images. At this stage, an almost perfect segmentation was achieved. In Table 2 are the evaluation metrics for the three cases. The network training performed by 1000 iterations per epoch for 100 epochs on a single GPU with a constant learning rate was 0.001. It is clear from Table 2 that as the number of training images increases, the segmentation quality increases up to perfect results, as in case 3. 

**Figure 9:**
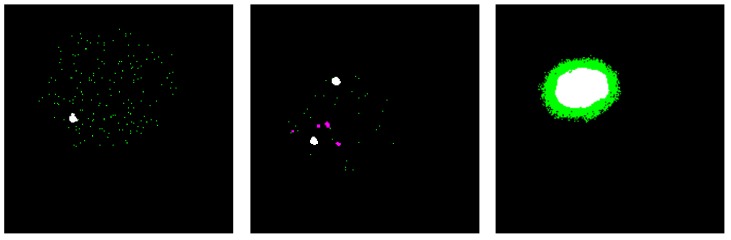
Samples of the resulting segmented image superimposed over the ground truth image. The correctly classified tumor pixels (known as true positive (TP)) are colored in white. The missed tumor pixels are colored in purple. The pixels that are predicted to belong to the tumor, but actually are pixels representing normal tissue or the background, are colored in green. The black color represents pixels that are correctly classified as normal or background.

For testing, a semantic segmentation is returned for the input image, with the classification scores for each categorical label. Figure 9 shows an illustration of the evaluation method, where the resulted segmented images are superimposed over the ground truth image. The correctly classified tumor pixels, known as true positive, are colored in white. It is clear from this figure that the results of the first are the one with the least accuracy, while the results of case 3 are perfect in terms of tumor detection; however, the tumor appears larger than it actually is.

The experimental results are presented in confusion matrices in Table 3, Table 4 and Table 5 for the test cases 1,2 and 3, respectively. The results displayed are normalized. 

In order to increase the insight on the presented results, Table 6 presents a comparison between the overall accuracy of the proposed method compared to some chosen work from the literature, according to the results reported in their papers. From this work, we have achieved higher accuracy than the work in the comparison. 

## 5. Conclusions

This paper presents experimental work to adopt a deep learning model, used for semantic segmentation of road scene understanding, for tumor segmentation in CT Liver scans in DICOM format.

SegNet is recent encoder–decoder network architecture that employs the trained VGG-16 image classification network as encoder, and employs corresponding decoder architecture to transform the features back into the image domain to reach a pixel-wise classification at the end. The advantage of SegNet over standard auto-encoder architecture is in the simple yet very efficient modification where the max-pooling indices of the feature map are saved, instead of saving the feature maps in full. As a result, the architecture is much more efficient in training time, memory requirements, and accuracy.

To facilitate binary segmentation of medical images, the classification layer was replaced with binary pixel classification layer. For training and testing, the standard 3D-IRCADb-01 dataset was used. The proposed method correctly detects most parts of the tumor, with accuracy above 86% for tumor classification. However, by examining the results, there were few false positives that could be improved by applying false positive filters or by training the model on a larger dataset.

As a future work, we propose using a new deep learning model as an additional level to increase the localization accuracy of the tumor, and hence reduce the FN rate and increase the IoU metric, like the work introduced in [20]. 

## Figures and Tables

**Table 1:** Terms used to define sensitivity, specificity, and accuracy.

		Predicted	
		Positive	Negative
**Actual**	**Positive**	TP	FN
	**Negative**	FP	TN

**Table 2:** Evaluation metrics for the training of the three test cases.

	# Training Images	Overall Ac.	Mean Ac.	Tumor Ac. (TPR)	Background Ac. (TNR)	Weighted IoU	F1-Score
**Case 1**	70	91.27%	93.43%	95.58%	91.27%	91.26%	21 91%
**Case 2**	124	93.36%	95.18%	97.34%	93.03%	92.99%	46.58%
**Case 3**	260	94.57%	97.26%	99.99%	94.52%	93.72%	62.16%

**Table 3:** Normalized confusion matrix for test case 1.

Case 1		Predicted	
		Tumor	Others
**Actual**	**Tumor**	45.82%	54.28%
	**Others**	0.31%	99.69%

**Table 4:** Normalized confusion matrix for test case 2.

Case 1		Predicted	
		Tumor	Others
**Actual**	**Tumor**	67.13%	32.87%
	**Others**	3.04%	96.95%

**Table 5:** Normalized confusion matrix for test case 3.

Case 1		Predicted	
		Tumor	Others
**Actual**	**Tumor**	99.99%	0%
	**Others**	5.48%	94.52%

**Table 6:** Comparison on the overall accuracy for the proposed method against other work in the literature.

Author (s)	Application	Method	Accuracy
Chlebus [11]	Liver tumor candidate classification	Random Forest	90%
Christ [12]	Automatic liver and tumor segmentation of CT and MRI	Cascaded fully convolutional neural networks (CFCNs) with dense 3D conditional random fields (CRFs)	94%
Yang [33]	Liver segmentation of CT volumes	A deep image-to-image network (DI2IN)	95%
Bi [7]	Liver segmentation	Deep residual network (Cascaded ResNet)	95.9%
Li [46]	Liver and tumor segmentation from CT Volumes	H-DenseUNet	96.5%
Yuan [32]	Automatic liver and tumor segmentation	Hierarchical Convolutional—Deconvolutional Neural Networks	96.7%
Wen Li et al. [28]	Patch-based liver tumor classification	Conventional Convolutional Neural Network (CNN)	80.6%
Our method	Liver tumor in CT Scans segmentation	Modified SegNet	98.8%
